# Impact of randomized blinded rechecking program on the performance of the AFB Microscopy Laboratory Network in Uganda: a decadal retrospective study

**DOI:** 10.1186/s12879-023-08406-6

**Published:** 2023-07-26

**Authors:** Andrew Nsawotebba, Ivan Ibanda, Dennis Mujuni, Susan Nabadda, Diana Nadunga, Joel Kabugo, Isa Adam, Enock Wekiya, Abdunoor Nyombi, Richard Nsubuga, Patrick Ademun, Kenneth Musisi, Fredrick Kangave, Moses Joloba

**Affiliations:** 1 Department of National Health Laboratory and Diagnostic Services, National Health Laboratory Services, Kampala, Uganda; 2 World Health Organisation Supranational Reference Laboratory - Uganda National TB Reference Laboratory, Kampala, Uganda; 3 Department of Pharmacology and Pharmacotherapeutics, School of Medicine and Surgery, King Ceasor University, Kampala, Uganda; 4 Department of Medical Microbiology, School of Biomedical Sciences, Makerere University, Kampala, Uganda

**Keywords:** AFB Microscopy, Randomized Blinded Re-Checking, Impact, Laboratory Network, External Quality Assurance, Lot Quality Assurance Sampling

## Abstract

**Background:**

Smear microscopy has remained the initial diagnostic test for presumptive tuberculosis (TB) patients in health facilities without the World Health Organization (WHO) recommended rapid diagnostic tools. In the Uganda TB laboratory network, the technique remains the only tool to monitor response to treatment among drug susceptible TB patients, with the country currently having over 1,600 microscopy TB testing units. It has been evidenced that acid-fast bacilli (AFB) microscopy’s yield highly depends on the staining technique and reading ability of the laboratory personnel. For the quality of TB testing in the country, the TB control program set up a Randomized Blinded Rechecking (RBRC) program in 2008 to monitor the testing performance of laboratories to continuously improve the reliability and efficiency of results. This is the first study to determine the effectiveness and impact of the RBRC program on the performance of the participating laboratories in Uganda.

**Methods:**

This was a retrospective cross-sectional study based on a record review of the RBRC’s annual results compilations between January 2008 and December 2017.

**Results:**

Between January 2008 and December 2017, a total of 265,523 smears were re-checked during the RBRC program. The number of enrolled laboratories in the RBRC program rose from 660 to 2008 to 1,406 in 2017. The RBRC program resulted in a statistically significant reduction in microscopy errors, with false positives decreasing from 12.8% to 2008 to 7.6% in 2017, false positive errors decreasing from 10 to 6.3%, false negative errors decreasing from 2.9 to 0.7%, quantification errors decreasing from 6.0 to 1.8%, and the overall sensitivity of smear microscopy compared to the controllers increased with statistical significance from 93 to 97%.

**Conclusion:**

The study reveals an overall significant error reduction and an improved sensitivity of smear microscopy upon continuous implementation of the RBRC program in an AFB microscopy TB laboratory network. Implementation of a RBRC program is crucial and essential to maintaining a reliable TB laboratory service that can facilitate accurate diagnosis and offset the disadvantages of using smear microscopy.

## Background

Tuberculosis (TB) is an old scourge that has remained captain among infectious diseases. Studies of human skeletons reveal that this public health menace has affected humans for thousands of years [1, 2]. It is currently the leading cause of death from a single infectious agent that was responsible for an estimated 1.5 million deaths in 2018 globally [2]. The Stop TB strategy emphasizes that tuberculosis control can be achieved if individuals with the disease receive accurate and timely diagnosis [3]. A network of laboratories that provide accurate, easily accessible, and reliable tuberculosis testing is essential to an efficient TB control strategy. [4, 5].

Despite the significant advancement in TB diagnostic and monitoring tools, classical smear microscopy remains the most common and economical method to detect pulmonary TB. The test of 135 years directly identifies Acid-Fast Bacilli (AFB) in Ziehl-Neelsen (ZN) sputum under a microscope [5–9]. This is because smear microscopy is simple, timely, inexpensive to adopt with minimal bio-safety and setup requirements [6, 7, 10, 11]. The purpose of smear microscopy is three-fold: To diagnose patients with infectious TB; monitor the treatment progress of individual patients; and, document cure at the end of treatment [4, 12]. The disadvantages, however, are that it is highly dependent on the quality of the sputum, the staining technique and reading ability of the laboratory personnel. Since its yield is highly dependent on the execution capacity of the scarce human resources who are routinely overloaded with reading large numbers of smears in the TB high burden countries, the reliability of laboratory results becomes a challenge [13]. If the laboratory diagnostic results are unreliable, then patients with infectious TB may not be diagnosed, resulting in continual transmission of the disease in the community and more severe disease in the individual [5]. On another hand, false-positive results could lead a patient placed on treatment unnecessarily, wasted medication, cause emotional trauma to patients and their families [5]. Errors in reading follow up smears can result in patients being placed on prolonged treatment or re-treatment, or premature treatment discontinuation [5].

To maintain a reliable laboratory service that can facilitate accurate diagnosis and offset the disadvantages of using smear microscopy, a well-functioning quality assurance (QA) system is essential to ensure that information generated by the laboratory is accurate, reliable and reproducible [5]. External Quality Assessment (EQA) is a key component of the QA program in laboratory networks [5, 11, 14]. Many clinical laboratory networks have successfully implemented various EQA programs and found them satisfactory in the identification of systematic problems in laboratories that were performing inadequately [10, 14–21].

The International Union Against Tuberculosis and Lung Disease (IUATLD)/ World Health Organization (WHO) recommended three EQA Programs for any Tuberculosis Laboratory Network [5]. Firstly, onsite evaluation to obtain a realistic picture of the conditions and practices of peripheral laboratories using standard checklists. Secondly, panel testing which involves sending stained and/or unstained smears from the reference laboratory to the peripheral laboratories to check individual’s proficiency in staining, reading and reporting; and lastly, randomized blinded rechecking [4]. The IUATLD and WHO, however, recommended RBRC as the most effective EQA program for monitoring the testing performance of individual laboratories to continuously improve the reliability and efficiency of AFB microscopy testing services [4, 5]. Randomized Blinded Rechecking (RBRC) involves monthly or quarterly collection of a sample of routine smears from participating microscopy centers for blinded re-reading at a designated EQA center with feedback to the microscopy center. Older guidelines that recommended rechecking all positive smears, 10% of negative smears and relied on one controller only, were found to be labor-intensive and inefficient for laboratory assessment [5]. One of the key recommendations of both the 1998 and 1999 IUATLD workshops in Bangkok and Madrid respectively, was to develop new guidelines to assist National Reference Laboratories (NRLs) in establishing (or implementing) and sustaining effective EQA programs for their local microscopy laboratories [5].

In 2002, with the support of IUATLD, WHO, Japan Anti-Tuberculosis Association (JATA), Koninklijke Nederlandse Chemische Vereeniging (KNCV), Centers for Disease Control and Prevention (CDC) and Association of Public Health Laboratories (APHL), a 14-member workgroup developed the multi-sponsored EQA guidelines which recommend Lot Quality Assurance Sampling (LQAS) for RBRC [5]. Lot Quality Assurance Sampling (LQAS) typically reflects the daily performance of a laboratory and also leads to small sample sizes in high volume sample settings, especially those with high positivity rates in routine smears [5]. This makes RBRC more efficient, feasible and affordable. The current guidelines also emphasize the need for performance assessments of EQA systems to evaluate their impact, detect performance problems, and success of problem-solving strategies [5].

In Uganda, all TB laboratories follow the WHO guidelines and grading system of microscopic diagnosis for all AFB smear microscopy readings (refer to Table 1 [6, 11].

**Table 1:** AFB smear microscopy grading system

Findings	Grade
No acid-fast bacilli found in at least 100 fields	Negative
1–9 acid-fast bacilli per 100 fields	Scanty (report exact figure/100)
10–99 acid-fast bacilli per 100 fields	1+
1–10 acid-fast bacilli per field in at least 50 fields	2+
More than 10 acid-fast bacilli per field in ≥ 20 fields	3+

A predetermined annual sample size of 40 slides per participating laboratory is expected to be sampled. The visiting District TB and Leprosy Supervisor (DTLS) therefore samples 10 slides per lab per quarter using a simple random sampling method (for the first slide to be picked) where slides are selected using the laboratory register and not directly from the slide box. The subsequent selection of slides is done serially following a calculated interval given as the quotient of the total number of slides processed in the register divided by 10 (the sample size per quarter). The details of each slide are recorded on a sampling form, including the unique identifier and peripheral results. The DTLS then gives the randomly sampled AFB smears to the District Laboratory Focal Person (DLFP), who is the first-level controller. The DTLS ensures that the DLFP has no access to the participating lab results; only a list of slide identification numbers and slides are given to the DLFP [6, 11]. Shortfalls such as those where the sampling is less or more frequent or visits were missed (e.g., due to unavailability of resources or other reasons) are made up for by collecting the required number of slides to reach the total annual sample size, i.e., by selecting more slides during the next visit.

**Fig. 1:**
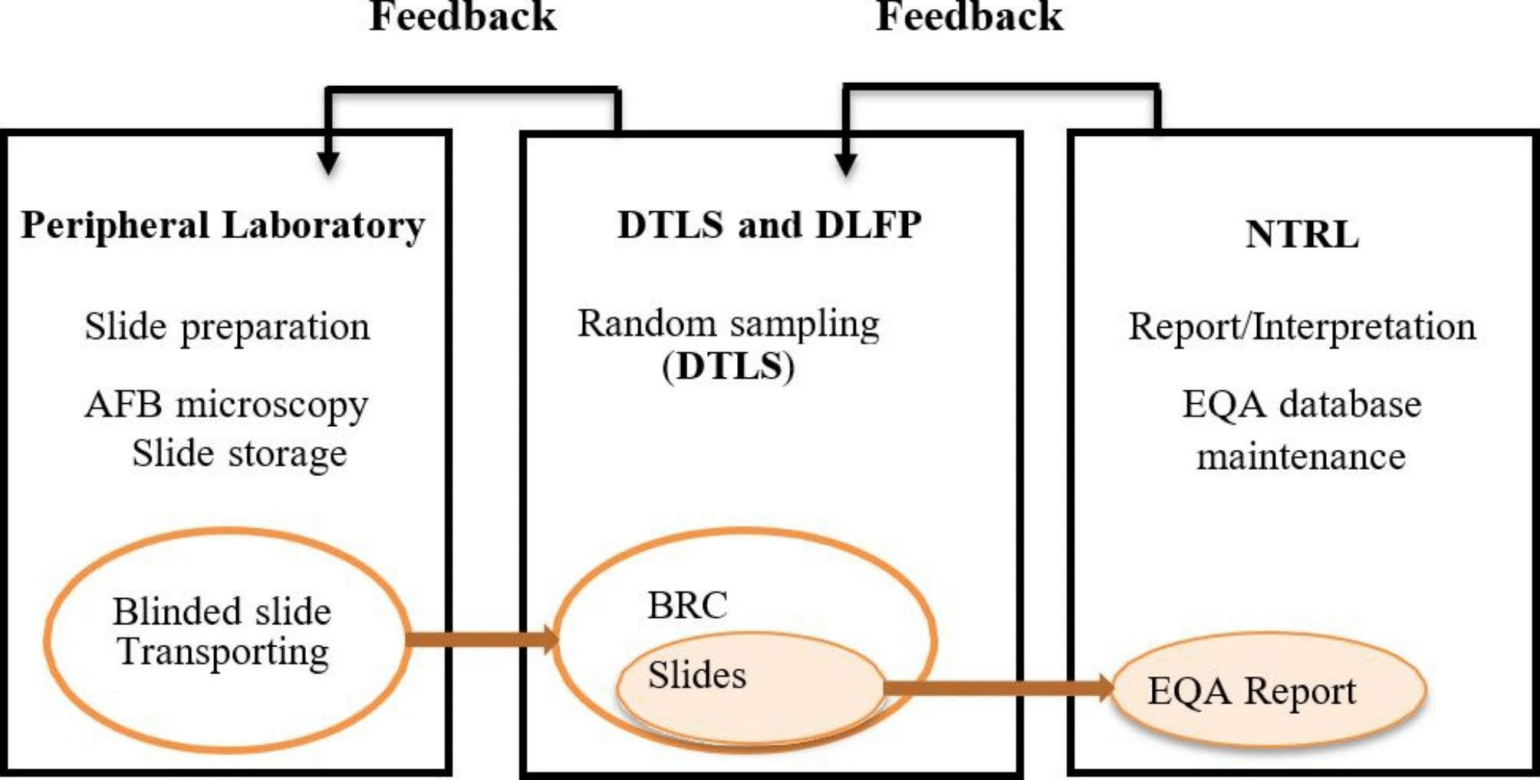
Algorithm of stepwise rechecking of the EQA system in Uganda DTLS: District TB and Leprosy Supervisor; DLFP: District Laboratory Focal Person; BRC: Blinded Rechecking

The smears are then blindly re-examined by the DLFP using the same microscopy technique as used in the peripheral laboratories. The DTLS then sends all the first-controlled smears (together with the forms of both peripheral and first controller results) to the Regional External Quality Assurance (REQA) Coordinator who is the second-level controller at National Tuberculosis Reference Laboratory (NTRL) as in Fig. 1.

Slides with discrepant results between the peripheral and first controller are re-examined by the REQA Coordinator using the same microscopy technique as used in the peripheral laboratories to exclude the presence of AFB with the highest possible probability and this result is considered the ‘gold standard’. The coordinator then compiles all results, identifying the types of errors and their origin (laboratory or first controller) as shown in Table 2 [6, 11].

**Table 2:** Classification of errors in AFB microscopy

Result of Technician	Result of Controllers				
	**Negative**	**1–9 AFB/100f**	**1+**	**2+**	**3+**
Negative	Correct	LFN	HFN	HFN	HFN
1–9 AFB/100f	LFP	Correct	Correct	QE	QE
1+	HFP	Correct	Correct	Correct	QE
2+	HFP	QE	Correct	Correct	Correct
3+	HFP	QE	QE	Correct	Correct

LFP: Low false-positive, HFP: High false-positive, LFN: Low false-negative, HFN: High false-negative, QE: Quantification error, f: field(s)

Since 2002 when IUATLD published the guidebook of EQA for AFB microscopy, little research on the evaluation of the new LQAS RBRC EQA system’s impact and implementation barriers in TB microscopic laboratories has been conducted in resource-limited countries [22]. Even the few studies that have been conducted evaluated RBRC data from few participating laboratories over a small period of time [13, 20, 23].

An efficient strengthened RBRC EQA system is an essential tool to the country’s TB control program with a role to monitor the testing performance of individual microscopy laboratories to continuously improve the reliability and efficiency of the microscopy TB results [5, 13, 20]. No operational research has been done to examine the actual functionality of the AFB blinded rechecking since its establishment as a national EQA program. This would demonstrate its effectiveness and impact. Additionally, it would quantify implementation and performance issues, examine their causes and associated limits, and ultimately identify areas for improvement in this important EQA program. Our study was aimed at evaluating the impact of the RBRC EQA Program in Uganda on the possible errors and diagnostic accuracy of the system.

## Methods

### Study design

This was a cross-sectional retrospective study. Quarterly AFB smear microscopy data and the corresponding EQA reading results collected from all the microscopy TB laboratories enrolled on RBRC EQA program between the first quarter of 2008 and the fourth quarter of 2017 were extracted from the NTRL AFB smear microscopy EQA databases. Database entries were verified against the paper-based archived RBRC sampling forms and reports at the National Tuberculosis Reference Laboratory (NTRL) of Uganda.

### Approach to obtaining data

The data used in this study was retrieved from the several compilations made at the various district/regional levels from 2008 to 2017 for a ten-year review.

### Data processing, analysis and interpretation

The results of the EQA randomized blinded re-checking system were evaluated based on the proportion of high false-positive (HFP), high false-negative (HFN), low false-positive (LFP), low false-negative (LFN), and quantification errors (QE). HFN; this is an error declared for a negative result of the laboratory and/or first controller versus a clearly positive result (1+, 2+, 3+) by a second controller. HFP; this is an error declared for a positive result of the laboratory and/or first controller versus a clearly negative result by a second controller. LFN; this is an error declared for a negative result of the laboratory and/or first controller versus a scanty result by a second controller. LFP; this is an error declared for a scanty positive result of the laboratory and/or first controller versus a clearly negative result by a second controller. QE; this is an error declared when there is more than 2 steps difference in quantification of positive results between the laboratory and/or first controller by the second controller.

Sensitivity, specificity, positive predictive value (PPV) and negative predictive value (NPV) were calculated to demonstrate the AFB smear reading performance of the participating laboratories relative to the final EQA re-checking (by the second controller) results as explained elsewhere [19]. Relative sensitivities were calculated by applying error rates found in the rechecking sample to the total positive and negative smear results that were reported in routine work, as explained elsewhere [6, 11]. This eliminated bias caused by the significantly different routine prevalence of positives in the different peripheral laboratories and between these and the samples presented to the controllers. Positive and negative predictive values (PVs) were calculated to provide discriminative power for false-positive and false-negative error analysis. All inferential tests were performed in Statav15. The Shapiro-Wilk test for normality was conducted prior to the regression analyses. Regression analyses to test for linear trends between the study parameters such as error and the year of detection were executed to determine whether the yearly percentage changes, that is, the declines in errors or rises in accuracy parameters, were statistically significant. *P* values less than 0.05 were considered to be statistically significant. Results were explained using absolute numbers and/or percentages. The frequencies/percentages of the different test results were extracted and presented in both tabular and graphical formats.

## Results

### Participation of laboratories in the EQA program for the 10 years

**Fig. 2:**
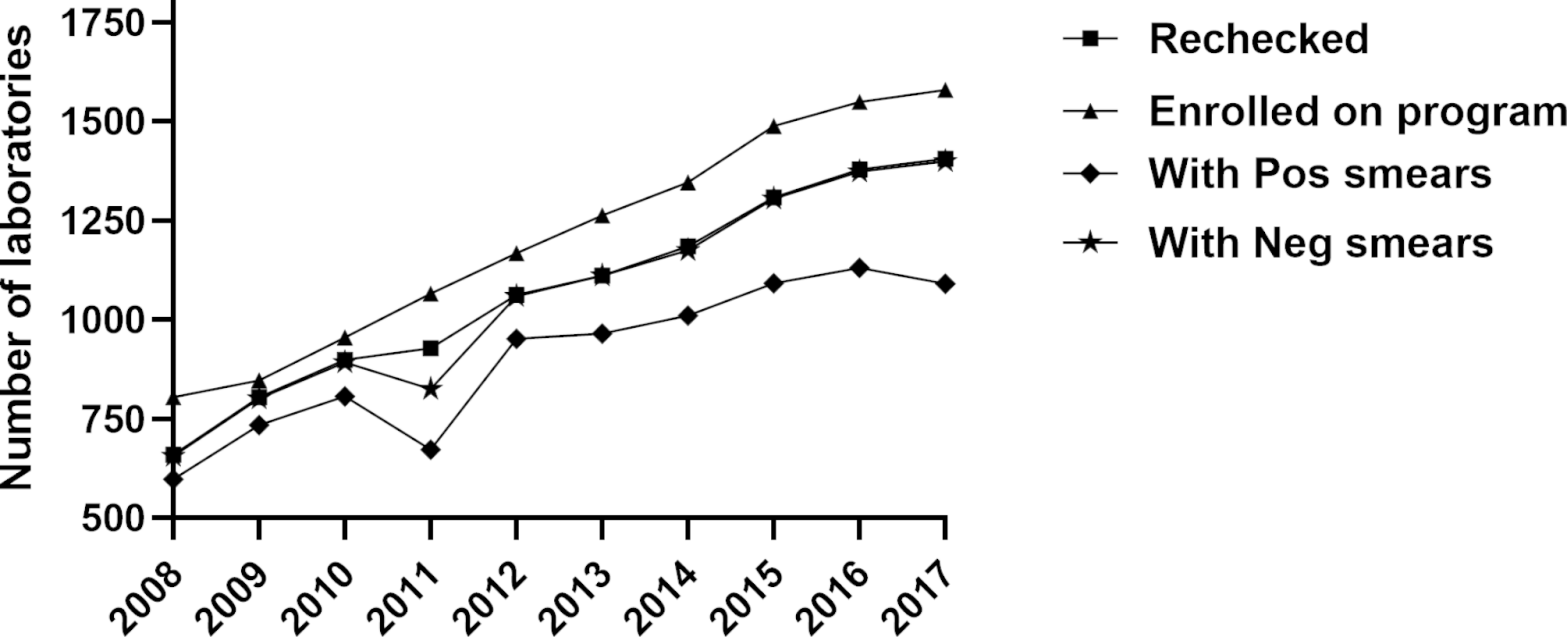
Laboratories enrolled and rechecked during the RBRC program between 2008 and 2017

Between 2008 and 2017, the laboratories enrolled for the EQA program increased from 660 to 1405, respectively. The numbers of laboratories enrolled and those rechecked during the random blinded rechecking program steadily increased over time (Fig. 2).

### The outcomes of the RBRC program implementation

**Fig. 3:**
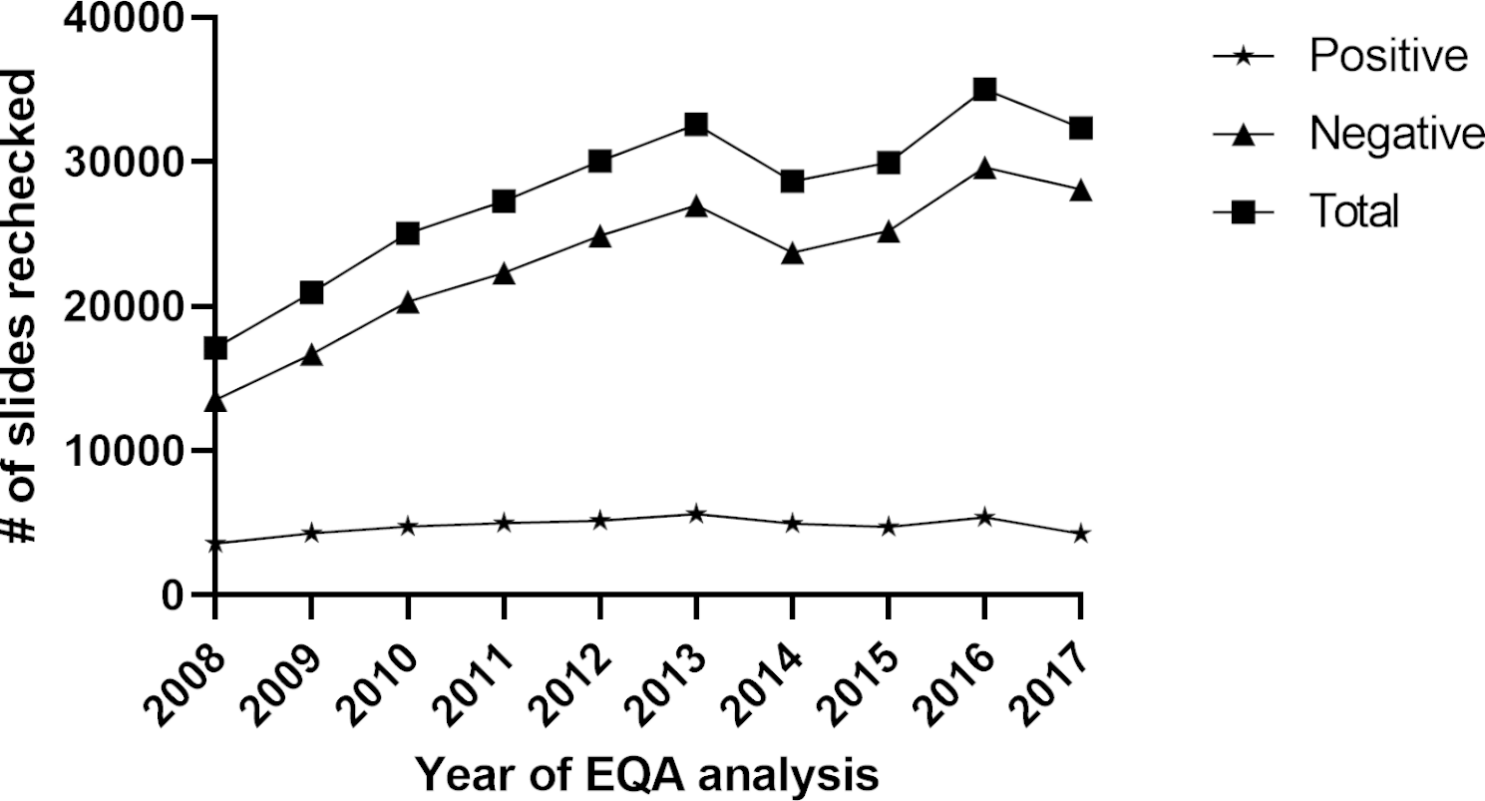
Number of slides rechecked during the RBRC program from 2008 through 2017

The number of slides rechecked was lowest in 2008 (17,1097) and highest in 2016 (35,021), see Fig. 3. Between 2008 and 2017, a total of 278,977 slides (47,661 positives and 231,316 negatives) were rechecked with a general slight increase each year. The number of positive slides rechecked was more less constant with no clear general trend as the numbers fluctuated from 3,597 to 2008, increased to 5,604 in 2013, then decreased thereafter to 3,529 in 2017 (with the exception 2016). On the other hand, the negative slides rechecked had a similar trend as the total number of slides as shown in Fig. 3.

The proportion of slides with scanty numbers of AFB bacilli, defined as the presence of 1–9 AFB in 100 fields, was highest in 2008 (8%) and decreased to 4% in 2016, though after that it slightly increased by 2% in 2017 (Table 3). This denoted a general decrease in the number of slides found with scanty AFB bacilli. The years 2009 and 2011 registered the lowest (55%) and highest (83%) proportions of labs, respectively, that correctly reported 100% of their positive slides as positive. The initial proportion (73% in 2008) of laboratories reporting at least 85% of their positives correctly improved over the years of the EQA RBRC implementation. There was a general decline in the proportion of laboratories with at least 1 high-false positive result reported, from 12% (2008) to 4% (2017). See Table 3.

The percentage of laboratories that accurately reported at least 85% of their negative slides was 64% in 2008; which progressively rose between 2009 (52%) and 2011 (88%); and following 2012, there was just a minor increase from 81% (2013) to 87% (2017). Furthermore, the proportion of laboratories that reported slides with at least one false negative steadily decreased from 12% to 2008 to 3% in 2017 (Table 3).

**Table 3:** The number of laboratories that participated in the program and details of randomized blinded rechecking exercise

Test result	Year of EQA analysis									
	**2008**	**2009**	**2010**	**2011**	**2012**	**2013**	**2014**	**2015**	**2016**	**2017**
Labs rechecked, % (n) Labs with P slides rechecked, n (%) Labs with N slides rechecked, n (%) Positive slides rechecked Scanty slides, % Labs with 100% TP, % (n) Labs with ≥ 85% TP, % (n) Labs with > 1 HFP, % (n) Negative slides rechecked Labs with ≥ 85% TN, % (n) Labs with > 1 FN, % (n) Total of slides rechecked, n	82 (660) 598 (90.6) 656 (99.4) 3598 8 61 (365) 73 (437) 12 (72) 13,511 64 (420) 12 (77) 17,109	95 (805) 734 (91.2) 802 (99.6) 4268 7 55 (404) 78 (573) 9 (66) 16,673 52 (417) 11 (87) 20,941	94 (899) 807 (89.8) 893 (99.3) 4745 7 70 (565) 81 (654) 8 (65) 20,305 76 (679) 13 (116) 25,050	87 (829) 673 (81.2) 825 (99.5) 4977 7 83 (559) 87 (586) 3 (20) 22,313 88 (726) 6 (49) 27,290	91 (1063) 952 (89.6) 1059 (99.6) 5160 7 66 (628) 80 (762) 6 (57) 24,877 81 (858) 9 (91) 30,037	88 (1112) 965 (86.8) 1112 (100.0) 5604 5 62 (598) 80 (772) 7 (68) 26,990 84 (934) 4 (48) 32,594	88 (1185) 1011 (85.3) 1175 (99.2) 4937 5 68 (687) 80 (809) 6 (61) 23,718 86 (1010) 4 (47) 28,655	88 (1309) 1092 (83.4) 1305 (99.7) 4729 5 67 (732) 82 (895) 5 (55) 25,215 87 (1135) 3 (37) 29,944	89 (1379) 1131 (82.0) 1374 (99.6) 5390 4 65 (735) 80 (905) 6 (68) 29,631 86 (1182) 3 (46) 35,021	89 (1405) 1091 (77.7) 1400 (99.6) 4254 6 74 (807) 85 (927) 4 (44) 28,082 87 (1218) 3 (36) 32,336

n – Number; % – Percentage; TP – True Positive; TN – True Negative FN; False Negative; HFP; High False Positive

### Evaluation of the impact of the rechecking program

**Fig. 4:**
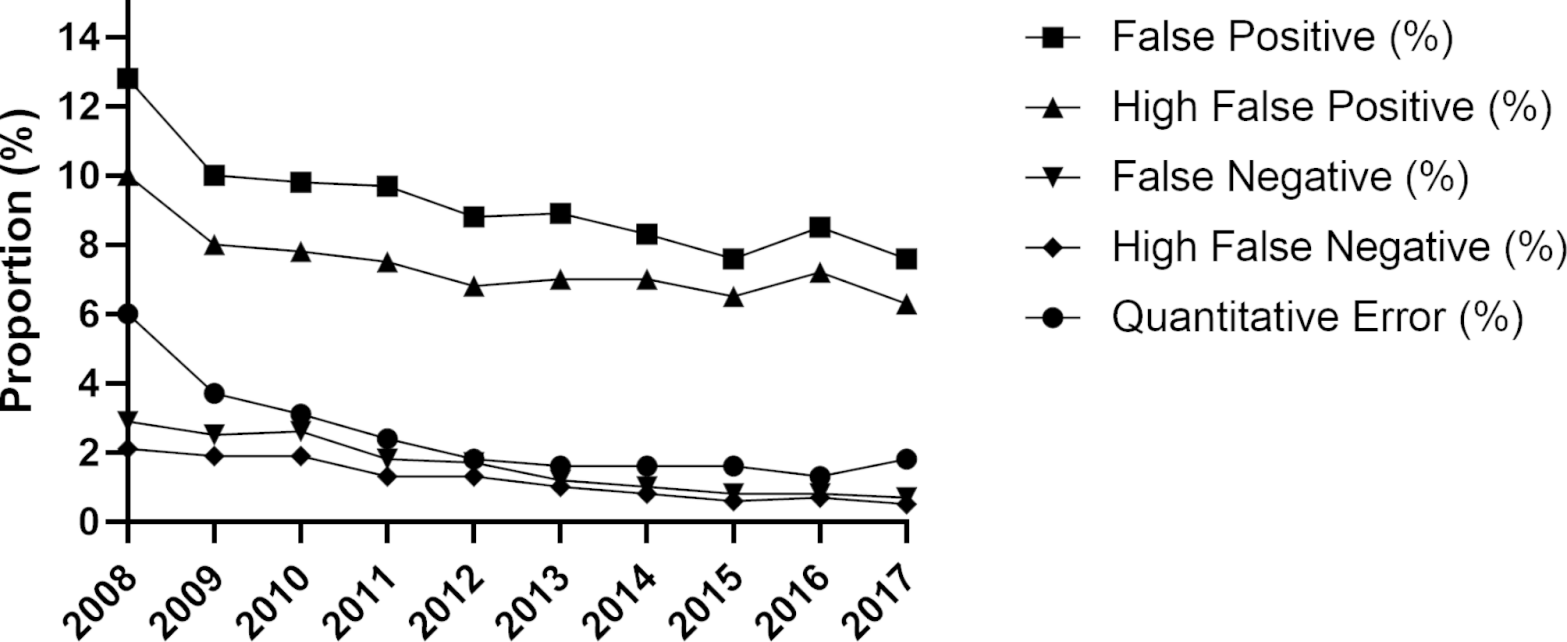
Trend analysis of the errors identified during the program for the entire laboratory network

The percentage of errors identified decreased while the diagnostic accuracy parameters increased with more years of participation in the RBRC EQA program for the laboratories (660) that were enrolled on the EQA program throughout the 10 years of analysis, see Table 4. False positives and high false positives steadily decreased from 12.8% to 10.0% in 2008 to 8.6% and 7.6% in 2017, respectively. Throughout the ten years of analysis, a similar pattern was observed in the percentages of false positives and high false positives recorded for the entire laboratory network (Fig. 4). On the other side, the false negatives, high false negatives and quantitative errors reported by the 660 labs gradually decreased from 2.9%, 2.1% and 6.0% in 2008 to 0.7%, 0.6% and 1.7% in 2017, respectively. Just as the false positives, there was a similar trend in the percentages of the false negative, high false negatives and quantitative errors for the entire laboratory network enrolled on the EQA program (See Fig. 4). The dropping trends in the above percentages over the ten years were found to be statistically significant with all the p values from the regression analysis, apart from that of the high false positives, found to beless than 0.05 (Table 4).

**Table 4:** Analysis of the impact of RBRC on the laboratories with a complete 10-year follow-up

Test parameter	Year of EQA analysis										*P* value
	**2008**	**2009**	**2010**	**2011**	**2012**	**2013**	**2014**	**2015**	**2016**	**2017**	
False Positive (%) High False Positive (%) False Negative (%) High False Negative (%) Quantitative Error (%) Sensitivity Positive Predictive Value Specificity Negative Predictive Value	12.8 10.0 2.9 2.1 6.0 89.0 87.2 96.6 97.1	10.2 8.3 2.5 1.9 3.7 90.3 89.8 97.4 97.5	9.8 7.6 2.7 2.0 3.2 88.5 90.2 97.7 97.3	8.5 5.9 1.8 1.3 2.5 91.7 91.5 98.1 98.2	9.5 7.6 2.0 1.6 2.2 90.4 90.5 98.0 98.0	8.9 7.3 1.4 1.2 2.1 93.3 91.1 98.1 98.6	8.2 7.0 1.1 0.8 1.9 94.7 91.8 98.2 98.9	7.9 6.6 0.8 0.6 1.6 95.8 92.1 98.4 99.2	10.2 8.7 0.8 0.7 1.5 95.5 89.8 98.0 99.2	8.6 7.6 0.7 0.6 1.7 95.0 91.4 98.7 99.3	**< 0.048** 0.344 **< 0.001** **< 0.001** **0.002** **< 0.001** **0.048** **0.002** **< 0.001**

With the exception of the HFP, all regression models that show a linear relationship in the trend of errors have P values below 0.05, thus we can say that there is a statistically significant linear relationship between the years and the reduction in the number of errors recorded during the implementation of the RBRC program

Sensitivity and specificity of the smear readings (random blinded rechecking) as an EQA program increased over the years from 90% to 97% in 2008 to 93% and 99% in 2017 for the entire network, respectively. The corresponding positive predictive values also increased from 88 to 95% while the negative predictive values were found to increase from 97% to d 99% in 2008 and 2017, respectively. It can be realized that similar trends were observed for all the 4 validity parameters even within the subset of the 660 laboratories with complete follow-up (Table 4).

## Discussion

Sputum smear microscopy continues to play a significant role in the diagnosis of *Mycobacteriumtuberculosis* (MTB) mainly in developing countries at peripheral testing sites without molecular WHO-recommended Rapid Diagnostic (mWRD) tools such as GeneXpert. The method serves as a GeneXpert substitute that is less expensive while simultaneously being useful for drug-sensitive tuberculosis treatment monitoring. In this study, we have demonstrated the effectiveness and impact of the RBRC program on the performance of the participating laboratories. Recent field studies that have been authored have shown how much these improved EQA program evaluations can actually affect a country’s ability to meet core competencies like those related to the detection of events with global public health significance while also fostering vivid confidence in the advancement of diagnostic network services in Africa [23].

The high frequency of HFN at the beginning of the EQA program in 2008 compared to the lowest observed at the end of the study in 2017 could be attributed to a number of factors including; poorly maintained microscopes, inexperienced smear readers, and inadequate training. Additionally, poor quality of staining technique and the lack of internal quality control have been reported to cause such errors [24, 25].

The Ugandan National TB Reference Laboratory (NTRL) evaluated the network of TB microscopy laboratories prior to the launch of the EQA program, taking into account the kind of equipment, human resources that were available, and their competency [26]. The consequent adjustments that were implemented following the assessment can be credited for the reported decrease in the frequency of labs with false positives and false negatives with sustained program participation in the years following 2008. These include, but are not limited to, providing refresher trainings, switching monocular microscopes for binocular ones, and promoting the recruitment of more qualified personnel [26]. A series of smear microscopy and subsequent refresher trainings were conducted in all regional laboratories and emphasis was put on quality of smears including their size, thickness, evenness, plus the staining technique and use of regular internal controls. These strategies have been recommended elsewhere as means of strengthening the laboratory external quality assurance system [27]. This could have enhanced the quality of smears as other studies have shown for other diseases [28], and for TB testing [29], thereby potentially increasing the accuracy and reliability of AFB microscopy.

Though the initial values for sensitivity (90%) and specificity (97) were high for the randomized blinded rechecking in 2008, these significantly increased over the ten years of implementation to 93% and 99%, respectively. According to the international standards, the ultimate values for sensitivity and specificity as determined in this study are unarguably high [5].

The positive and negative predictive value (s) increased with more years of participation in the EQA program given that these correspond with the decreased frequency of high false positives and false negatives. Additionally, the continued participation in the program potentially consolidated the competencies of AFB smear readers at the diagnostic sites over the years, explaining the gradual drop in the error trends within our study period. We recently demonstrated that continued participation in external quality assurance programs improves tuberculosis testing accuracy of laboratories [30].

The RBRC program, in general, had a favorable impact on the national TB laboratory microscopy network, increasing the sensitivity and specificity of the use of microscopes for accurate tuberculosis diagnosis. The rechecking program presented in this paper was simultaneously accelerated by; intensive support supervisions, the establishment of laboratory quality management system (LQMS), the availability of sufficient resources that compelled laboratories to ensure enough supplies in order to achieve quality testing, improved identification and management of nonconformities within the microscopy sections (path of workflow), preventive action practice, and continuous quality improvement. These efforts, the majority of which have been asserted elsewhere [31], ultimately improved internal training, competence evaluation, and staff performance reviews, equipment (maintenance, servicing, and repair) and reagents management, use of process controls that necessitates testing of quality controls, development and use of microscopy standard operating procedures (SOPs), and enrollment of many participating laboratories onto Stepwise Laboratory Management Towards Accreditation (SLMTA), some of which were subsequently accredited to International Organization for Standardization (ISO) standards.

Our experience in implementing the RBRC has allowed us to appreciate how affordable it is when compared to other EQA methods such as proficiency testing and on-site supervisions, in terms of intensive planning that requires time, resources and capacity building needs among others. This is stated with the background that microscopy as a diagnostic method in itself is simple, widely available at diagnostic sites [32], inexpensive and does not require sophisticated training or establishment although it does require a very good system of quality assurance [15].

## Conclusions

Our study commends that the RBRC EQA program is effective and its continuous implementation commensurate with reduced errors, high sensitivity, and specificity of the laboratories employing smear microscopy, as this is evidenced by the increased improvement rates. These findings are reasonable enough to deem RBRC as an essential component of the EQA program. The need to evaluate the impact of the RBRC program after decentralization must be advocated so as to ascertain the effectiveness of the move on AFB External Quality Assurance of smear microscopy.

## Data Availability

The datasets used and/or analyzed during the current study are available from the corresponding author on reasonable request.

## Abbreviations

AFB: Acid-Fast Bacilli
APHL: Association of Public Health Laboratories
DLFP: District Laboratory Focal Person
DTLS: District TB and Leprosy Supervisor (DTLS)
EQA: External Quality Assessment
GLRA: German Leprosy and TB Relief Association
HFN: High False Negative
HFP: High False Positive
ISO: International Organization for Standardization
IUATLD: International Union Against Tuberculosis and Lung Disease
JATA: Japan Anti-Tuberculosis Association
KNCV: Koninklijke Nederlandse Chemische Vereeniging
LFN: Low False Negative
LFP: Low False Positive
LQAS: Lot Quality Assurance Sampling
LQMS: Laboratory Quality Management System
MTB: Mycobacterium Tuberculosis
mWRD: molecular WHO-recommended Rapid Diagnostic
NGO: Non-Governmental Organizations
NPV: Negative Predictive Value
NRL: National Reference Laboratories
NTRL: National Tuberculosis Reference Laboratory
PPV: Positive Predictive Value
PV: Predictive Value
QA: Quality Assurance
QE: Quantification Error
RBRC: Randomized Blinded Rechecking
REQA: Regional External Quality Assurance
SLMTA: Stepwise Laboratory Management Towards Accreditation
SOP: Standard Operating Procedures
TB: Tuberculosis
USAID: United States Agency for International Development
WHO: World Health Organization
ZN: Ziehl – Neelsen
